# Antioxidant preconditioning improves therapeutic outcomes of adipose tissue-derived mesenchymal stem cells through enhancing intrahepatic engraftment efficiency in a mouse liver fibrosis model

**DOI:** 10.1186/s13287-020-01763-y

**Published:** 2020-06-16

**Authors:** Naishun Liao, Yingjun Shi, Yingchao Wang, Fangyu Liao, Bixing Zhao, Youshi Zheng, Yongyi Zeng, Xiaolong Liu, Jingfeng Liu

**Affiliations:** 1 The United Innovation of Mengchao Hepatobiliary Technology Key Laboratory of Fujian Province, Mengchao Hepatobiliary Hospital of Fujian Medical University, Fuzhou, 350025 People’s Republic of China; 2 Liver Disease Center, The First Affiliated Hospital of Fujian Medical University, Fuzhou, 350007 People’s Republic of China; 3 Mengchao Med-X Center, Fuzhou University, Fuzhou, 350116 People’s Republic of China; 4 The Liver Center of Fujian Province, Fujian Medical University, Fuzhou, 350025 People’s Republic of China

**Keywords:** Adipose tissue-derived mesenchymal stem cells, Reactive oxygen species, Liver fibrosis, Cell engraftment efficiency, Stem cell therapy

## Abstract

**Background:**

Although it has been preclinically suggested that adipose tissue-derived mesenchymal stem cell (ADSC)-based therapy could effectively treat chronic liver diseases, the hepatic engraftment of ADSCs is still extremely low, which severely limits their long-term efficacy for chronic liver diseases. This study was designed to investigate the impact of antioxidant preconditioning on hepatic engraftment efficiency and therapeutic outcomes of ADSC transplantation in liver fibrotic mice.

**Methods:**

Liver fibrosis model was established by using intraperitoneal injection of carbon tetrachloride (CCl_4_) in the male C57BL/6 mice. Subsequently, the ADSCs with or without antioxidant pretreatment (including melatonin and reduced glutathione (GSH)) were administrated into fibrotic mice via tail vein injection. Afterwards, the ADSC transplantation efficiency was analyzed by ex vivo imaging, and the liver functions were assessed by biochemical analysis and histopathological examination, respectively. Additionally, a typical hydrogen peroxide (H_2_O_2_)-induced cell injury model was applied to mimic the cell oxidative injury to further investigate the protective effects of antioxidant preconditioning on cell migration, proliferation, and apoptosis of ADSCs.

**Results:**

Our data showed that antioxidant preconditioning could enhance the therapeutic effects of ADSCs on liver function recovery by reducing the level of AST, ALT, and TBIL, as well as the content of hepatic hydroxyproline and fibrotic area in liver tissues. Particularly, we also found that antioxidant preconditioning could enhance hepatic engraftment efficiency of ADSCs in liver fibrosis model through inhibiting oxidative injury.

**Conclusions:**

Antioxidant preconditioning could effectively improve therapeutic effects of ADSC transplantation for liver fibrosis through enhancing intrahepatic engraftment efficiency by reducing oxidative injuries. These findings might provide a practical strategy for enhancing ADSC transplantation and therapeutic efficiency.

## Background

Chronic liver diseases are typically characterized by the intrahepatic inflammation, oxidative stress and development of liver fibrosis, and eventually leading to liver cirrhosis [1]. Liver transplantation is the most effective strategy for treating end-stage liver diseases including liver cirrhosis, but it is suffered from shortage of donated livers in real clinical practice. Regenerative medicine using adipose tissue-derived mesenchymal stem cell (ADSC) transplantation provides a promising alternative strategy for chronic liver disease treatment by using the intrinsic abilities of ADSCs including multilineage differentiation potential, self-renewal, and immunomodulation properties. In particular, ADSC transplantation has been widely used for treating liver fibrosis [2], non-alcoholic liver diseases [1, 3–5], and cirrhosis [6] in the preclinical studies, but the long-term therapeutic outcomes are very poor and far from satisfaction, due to the extremely low hepatic engraftment efficiency of ADSCs. In most cases, the hepatic retention of stem cells is less than 5% after cell infusion for 4 weeks [7]. Therefore, there is an urgent need to improve the intrahepatic engraftment efficiency of ADSCs to accelerate the clinical translation of ADSC-based therapies for treating chronic liver diseases. Although it has been reported that instantaneous irradiation or vasodilators (e.g., nitroglycerin and phenytolamine) could enhance cell transplantation efficiency by increasing hepatic vascular permeability in animal models [8–12], these methods also bring severe adverse effects including additional hepatocellular injuries and uncontrollable internal bleeding, therefore significantly hindering their applications in real clinical practice.

It has been widely acknowledged that most transplanted cells were dead in the first few days post-transplantation owing to the oxidative stress induced by reactive oxygen species (ROS) at the site of injury [13]. Many key biological features of ADSCs could be significantly affected by ROS-induced oxidative injuries, including cell proliferation, adhesion, and migration, as well as multilineage differentiation and self-renewal abilities both in vitro and in vivo [14–18], and this might be a main reason for low engraftment efficiency of ADSCs after transplantation. Therefore, reducing ROS-induced cell injury during ADSC transplantation might provide a novel and practical strategy to improve the hepatic engraft efficiency for chronic liver disease treatment.

Previous studies have suggested that antioxidant pretreatment could improve cell survival and enhance ADSC therapeutic effects for myocardial infarction [19, 20]. In addition, it has also been suggested that antioxidant preconditioning could preserve stemness of mesenchymal stem cells [21, 22]. However, whether antioxidant preconditioning could improve the hepatic engraft efficiency and enhance therapeutic outcomes of ADSCs for chronic liver diseases as well as the underlying molecular mechanisms has not yet been investigated. In this study, the hepatic engraft efficiency and therapeutic effects of antioxidant-preconditioned ADSCs on CCl_4_-induced liver fibrosis mice, as well as the potential protective mechanism of antioxidant preconditioning, were fully investigated. Our results suggested that antioxidant preconditioning with GSH or melatonin could be used to enhance intrahepatic retention and therapeutic effects of ADSC transplantation for liver fibrosis. Mechanistically, the results also showed that antioxidant preconditioning could promote cell migration and proliferation, and inhibit cell apoptosis in ROS-induced cell injuries.

## Methods

### Animals

Eighty-five adult male C57BL/6 mice (4 weeks, weight 18–20 g) were obtained from the Shanghai Slack Laboratory Animal Center (license no. SCXK hu 2017-0005). All animals were housed in standard laboratory barrier facilities at 22–25 °C with a standard 12/12 light-dark cycle and 55–60% relative humidity. The mice had ad libitum access to food and autoclaved water. All animal procedures were approved by the Animal Ethics Committee of Mengchao Hepatobiliary Hospital of Fujian Medical University and Fuzhou General Hospital (Fuzhou, China).

### Isolation and culture of ADSCs

Isolation of ADSCs was performed according to our previous reports [2–4]. Briefly, subcutaneous fat tissues were collected from the groin area of male C57BL/6 mice (*n* = 10), and then cut into about 0.1 mm^3^ size and digested with 0.1% type I collagenase (Sigma-Aldrich, USA) in HBSS (Hyclone, USA) at 37 °C for 1 h. Afterwards, the digestive solutions were neutralized by α-MEM (Hyclone, USA) containing 20% FBS (Gibco, USA), filtered through a 100-μm cell strainer, followed by eliminating the red blood cells with osmotic lysates (Biyuntian Biological Co., Ltd., Shanghai, China). Finally, the cells were collected and seeded into T-75 flasks at a density of 1 × 10^6^/mL with complete medium (α-MEM containing 10% FBS). Once the cell confluence reached about 90%, they were detached using 0.25% trypsin–EDTA (Gibco, USA) and passaged at a ratio of 1:3. The ADSCs from passage 3 were applied for further usage.

### Antioxidant pretreatment

Antioxidants including GSH and melatonin were obtained from Aladdin Chemical (Shanghai, China). Each antioxidant was firstly dissolved in DMSO (Sigma-Aldrich, USA) to the concentration of 1 mM. For antioxidant pretreatment, the ADSCs were cultured with complete medium supplemented with 10 μM antioxidant since the first passage. All preconditioned cells were rinsed with PBS for three times to remove any residual antioxidant before further analysis.

### Cell labeling

For ADSC cell labeling, the ADSCs were re-suspended with 1 μM CellTracker™ Cm-dil (Qcbio Science&Technologies Co., Ltd., Shanghai, China) at a density of 1 × 10^6^/mL with complete medium at 37 °C for 3 min, followed by incubating at 4 °C for 9 min, and rinsed with PBS for three times, and finally collected for cell transplantation.

### Liver fibrosis model establishment and ADSC engraftment examination

Liver fibrosis model was established by intraperitoneal injection of 20% carbon tetrachloride (CCl_4_) with a dose of 5 mL/kg diluted in olive oil, 2 times per week, for 12 weeks. Following the development of liver fibrosis, which was verified by pathological assessment, the mice were divided into three groups: the control group (*n* = 15), mice with tail vein injection of ADSCs (1 × 10^6^ cells/mouse); the GSH group (*n* = 15), mice with tail vein injection of GSH-pretreated ADSCs (1 × 10^6^ cells/mouse); and the melatonin group (*n* = 15), mice with tail vein injection of melatonin-pretreated ADSCs (1 × 10^6^ cells/mouse). After ADSC transplantation for 1 h, 4 h, 1 day, 3 days, and 7 days, mice were sacrificed with 2% pentobarbital sodium (100 mg/kg; Sigma-Aldrich). Then, the major organs including the heart, liver, spleen, lung, and kidney were collected, and the distribution of ADSCs in these tissues was observed by using the IVIS system (PerkinElmer, USA). Liver tissues were collected to prepare paraffin section to further observe the hepatic engraftment efficiency of ADSCs using LSM780 confocal microscope (Zeiss, Germany).

### ADSC therapy for liver fibrosis

**Fig. 1:**
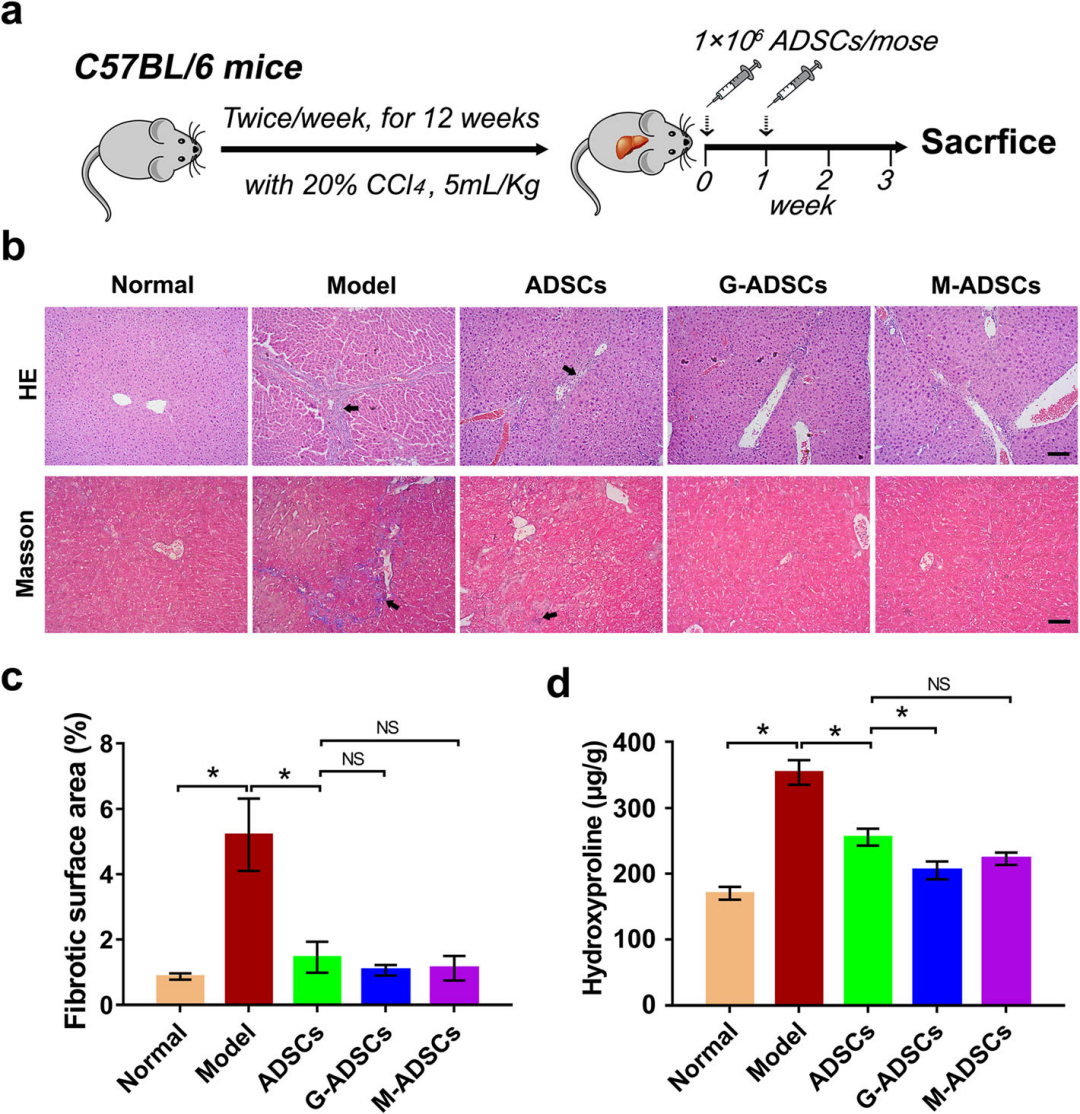
Antioxidant preconditioning enhances the tissue repair function of ADSC transplantation for liver fibrosis in vivo. **a** Schematic illustration of ADSC therapy in a murine model of liver fibrosis. **b** Representative images of liver sections stained with H&E and Masson’s trichrome after ADSC transplantation with or without antioxidant pretreatment (scale bar, 100 μm). **c** The fibrotic surface area of Masson’s trichrome positive stained liver sections (*n* = 6 per group; *p < 0.05). **d** Hydroxyproline content of liver tissues after ADSC transplantation with or without antioxidant pretreatment (*n* = 6 per group; *p < 0.05). ADSCs, adipose tissue-derived mesenchymal stem cells; G-ADSCs, ADSCs pretreated with reduced glutathione; M-ADSCs, ADSCs pretreated with melatonin

Twenty-four liver fibrosis mice were divided into four groups: the model group (*n* = 6), mice with tail vein injection of the same dose of normal saline (0.2 mL); the ADSC group (*n* = 6), mice with tail vein injection of ADSCs (1 × 10^6^ cells/mouse); the G-ADSC group (*n* = 6), mice with tail vein injection of GSH-pretreated ADSCs (1 × 10^6^ cells/mouse); and the M-ADSC group (*n* = 6), mice with tail vein injection of melatonin-pretreated ADSCs (1 × 10^6^ cells/mouse). Each group was treated with normal saline or ADSC transplantation once/week, for 2 weeks; after the last ADSC transplantation for 2 weeks, mice were sacrificed with 2% pentobarbital sodium (100 mg/kg; Sigma-Aldrich), and the liver tissues and sera were collected for further investigation (Fig. 1a).

### Biochemical assays of liver function

To determine whether antioxidant preconditioning could accelerate liver function recovery after ADSC transplantation, the serum levels of aspartate aminotransferase (AST), alanine aminotransferase (ALT), total bilirubin (TBIL), and albumin (ALB), as well as the hydroxyproline content in liver tissues, were analyzed by biochemical analysis using colorimetric assay kits (Nanjing Jiancheng Bioengineering Institute, Nanjing, China) according to the manufacturer’s instructions, respectively.

### Histological examination

Liver tissues were collected and fixed in 4% paraformaldehyde (PFA) for 24 h at room temperature, followed by the gradual dehydration of ethanol and embedding in paraffin. Then, the paraffin-embedded tissues were sectioned into slices. Afterwards, the tissue sections were stained by hematoxylin and eosin (H&E) for histological analysis. To further observe fibrotic changes in liver tissues, Masson’s trichrome staining was performed using a commercial kit (Nanjing Jiancheng Bioengineering Institute, Nanjing, China) according to the manufacturer’s protocol. The histological examination was independently performed by two pathological experts using an ortho-microscope (Zeiss, Germany), and the fibrotic area was analyzed using the ZEN 2012 Light Edition imaging analysis system (Zeiss, Germany).

### Cell viability assay

In order to clarify the mechanism of anti-ROS oxidative injury of antioxidant preconditioning on ADSCs, the typical H_2_O_2_-induced oxidative injury model was established as previously described [23] with slight modification. Briefly, antioxidant pretreated ADSCs (passage 3) were cultured in a 96-well micro-plate (Corning, USA) at a density of 1 × 10^4^ cells/well with 150 μL complete medium and incubated at 37 °C under 5% CO_2_ atmosphere for 24 h. After that, the cultured medium of each well was replaced by fresh complete medium supplied with 300 μM H_2_O_2_, and the cells incubated with complete medium were used as the negative control. After incubation for 24 h, the cell viability was assessed by using a CCK-8 cell proliferation kit (Dojindo Molecular Technologies, Tokyo, Japan) following the manufacturer’s protocol. Finally, the absorbance of each well at 450 nm was measured in a micro-plate reader (Spectra Max M5).

### Cell apoptosis assay

To determine the protective effect of antioxidant preconditioning on ADSC cell apoptosis, antioxidant pretreated ADSCs (passage 3) were cultured in a 6-well plate at a density of 1 × 10^5^ cells/well for 24 h, followed by the incubation with 300 μM H_2_O_2_ in fresh complete medium for another 24 h, and finally analyzed using an Annexin V-FITC apoptosis assay kit (Dojindo Molecular Technologies, Tokyo, Japan) in flow cytometry (Becton Dickinson, USA).

### Cell migration assay

Trans-well migration assay was use to explore the effects of antioxidant preconditioning on cell migration of ADSCs. Briefly, antioxidant-preconditioned ADSCs were treated with 300 μM H_2_O_2_ in fresh complete medium for 24 h, and then, the cells were collected; afterwards, 5 × 10^5^ treated cells were seeded into the upper compartment of the Trans-well units (Millipore, USA) in α-MEM containing 2% FBS, and the lower compartment was filled with complete medium. Twenty-four hours later, the filters were fixed with 4% PFA at room temperature for 20 min; then, the filters were washed with PBS for three times and stained with crystal violet at room temperature for 3 h; afterwards, the unmigrated cells were removed and the migrated cells on the lower surface of Trans-well units were observed using an inverted microscope (Zeiss, Germany) at a magnification of × 200. To further quantify the migration rate, we randomly selected five fields to count the number of migrated ADSCs.

### Western blot analysis

Cells were lysed with RIPA lysis buffer (0.5 M Tris-HCl, 10 mM EDTA, 1.5 M NaCl, 10% NP-40, 2.5% deoxycholic acid, pH = 7.4), and the protein concentration was quantified using a BCA assay kit (TransGen Biotech, Beijing, China). Afterwards, equal amounts of protein lysate were separated by 10% SDS-PAGE electrophoresis and transferred to nitrocellulose membranes (PALL, USA) in transfer buffer (96 mM glycine, 12 mM Tris base, pH 8.3, and 20% methanol). Then, the membranes were blocked for 2 h in the TBST buffer with 5% BSA and subsequently incubated with the C-X-C chemokine receptor type 4 (CXCR4), Bax, Bcl-2, and Cyclin-D1 (all from Wuhan Boster Biological Technology Co., Ltd., Wuhan, China; 1:500 dilution) at 4 °C overnight. Next, the membranes were washed with TBST buffer for three times and incubated with an anti-rabbit-conjugated secondary antibody (1:8000 dilution, Santa Cruz Biotechnology) for 1 h at room temperature. Finally, the protein expression levels were detected by an enhanced chemiluminescence system, and the quantitative analysis was performed using the Image J software.

### Statistical analysis

Data were expressed as the mean ± standard deviation (SD). All the statistical analyses were performed with GraphPad Prism version 7.0 (GraphPad Software, CA, USA). *T* test was used to assess the statistical analysis between the two groups. *p* < 0.05 was considered as statistically significant.

## Results

### Antioxidant preconditioning could enhance tissue repair functions of ADSC transplantation for liver fibrosis

To investigate the effect of antioxidant preconditioning on tissue repair functions of ADSC transplantation for liver fibrosis, the histological examination of the liver tissues was performed. As shown in Fig. 1b, the normal liver tissues did not exhibit any liver fibrosis and inflammation, while the typical fibrosis was clearly observed around the central vein of liver tissues of the model group, indicating the liver fibrosis model was successfully established by CCl_4_ inducing protocol; comparing with the model group, there was less evidence of liver fibrosis and inflammation in the ADSC transplantation groups, which means ADSC transplantation could reduce liver fibrosis; significantly, the inflammation and liver fibrosis in the GSH- or melatonin-pretreated ADSC group were much less than those in the normal ADSC treated group, and the hepatic morphology of the antioxidant-preconditioned ADSC groups was close to normal, suggesting that antioxidant preconditioning could increase ADSC repair function on pathological changes of liver fibrosis.

In order to further determine the degree of liver fibrosis, we also qualified the fibrotic area of Masson’s trichrome staining. As shown in Fig. 1c, the fibrotic area was significantly increased in the CCl_4_-induced liver fibrosis model compared with those in the normal mice, while the fibrotic area was significantly decreased after ADSC transplantation; of note, the decreased liver fibrosis was observed in the antioxidant-preconditioned ADSC groups compared with those in the conventional ADSC therapy group although there was no statistical difference, indicating that antioxidant preconditioning might improve the ability of ADSCs to inhibit the degree of liver fibrosis.

Based on the helpful effects of antioxidant preconditioning on ADSC transplantation for liver fibrosis, we next analyzed the hydroxyproline content in liver tissues. Compared with the normal group, the hepatic hydroxyproline content was significantly increased in the CCl_4_-induced liver fibrosis model group, while the content of hydroxyproline in the ADSC treated groups exhibited markedly decrease compared to those in the model group; significantly, lower hydroxyproline content was observed in the antioxidant-preconditioned ADSC groups compared with the un-pretreated ADSC group (Fig. 1d), which means that antioxidant preconditioning could enhance the ability of ADSCs to inhibit hydroxyproline production in fibrotic liver tissues.

### Antioxidant preconditioning could improve ADSCs’ ability to restore liver function in liver fibrosis mice

**Fig. 2:**
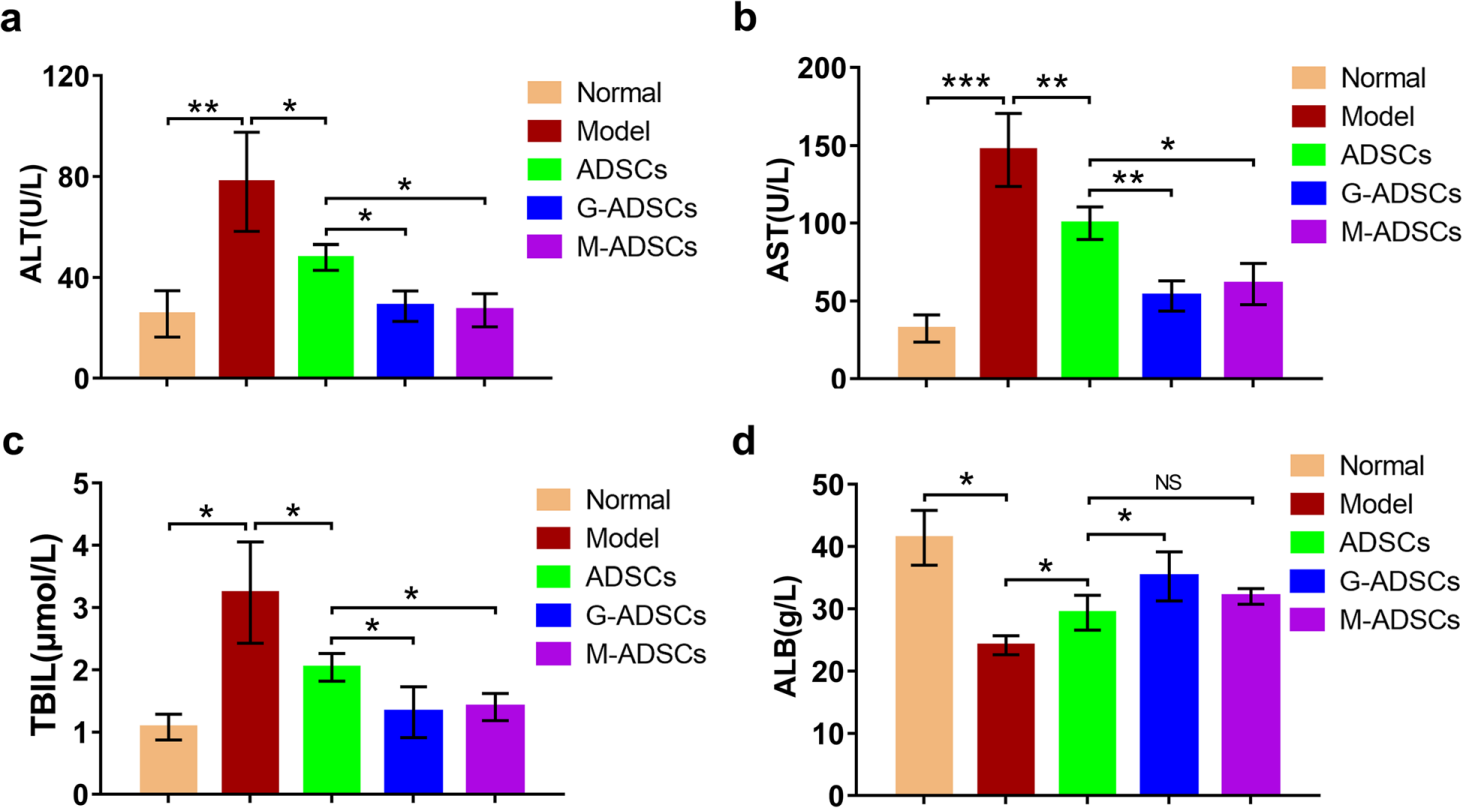
Antioxidant preconditioning enhances ADSC’s ability to restore liver function in vivo. The serum level of ALT (**a**), AST (**b**), TBIL (**c**), and ALB (**d**) after ADSC transplantation (*n* = 6 per group; *p < 0.05; **p < 0.01; ***p < 0.001). ADSCs, adipose tissue-derived mesenchymal stem cells; G-ADSCs, ADSCs pretreated with reduced glutathione; M-ADSCs, ADSCs pretreated with melatonin; ALB, albumin; ALT, alanine aminotransferase; AST, aspartate aminotransferase; TBIL, total bilirubin

We next measured the serum levels of AST, ALT, TBIL, and ALB to further confirm the liver functions of fibrotic mice after ADSC transplantation. As shown in Fig. 2, the markedly increased serum levels of AST, ALT, and TBIL and the decreased serum level of ALB were observed in the CCl_4_-induced liver fibrosis model comparing with those in the normal group, suggesting the serious hepatic dysfunctions in CCl_4_-treated mice; however, the reduced serum levels of AST, ALT, and TBIL and the increased ALB levels could be clearly observed in the ADSC therapy groups when compared with those in the model group, implying that the ADSC transplantation could recover the liver functions of liver fibrosis murine model; moreover, comparing with the normal ADSC group without preconditioning, the decreased serum levels of AST, ALT, and TBIL and the increased ALB level were more obvious in the GSH- or melatonin-pretreated ADSC group; significantly, the serum level of ALT and TBIL in the antioxidant-preconditioned ADSC groups was close to normal. Taken together, these results suggested that antioxidant preconditioning using GSH or melatonin could significantly enhance ADSCs’ ability to accelerate liver function recovery in liver fibrotic mice.

### Antioxidant preconditioning could enhance intrahepatic engraftment of ADSCs in liver fibrosis mice

**Fig. 3:**
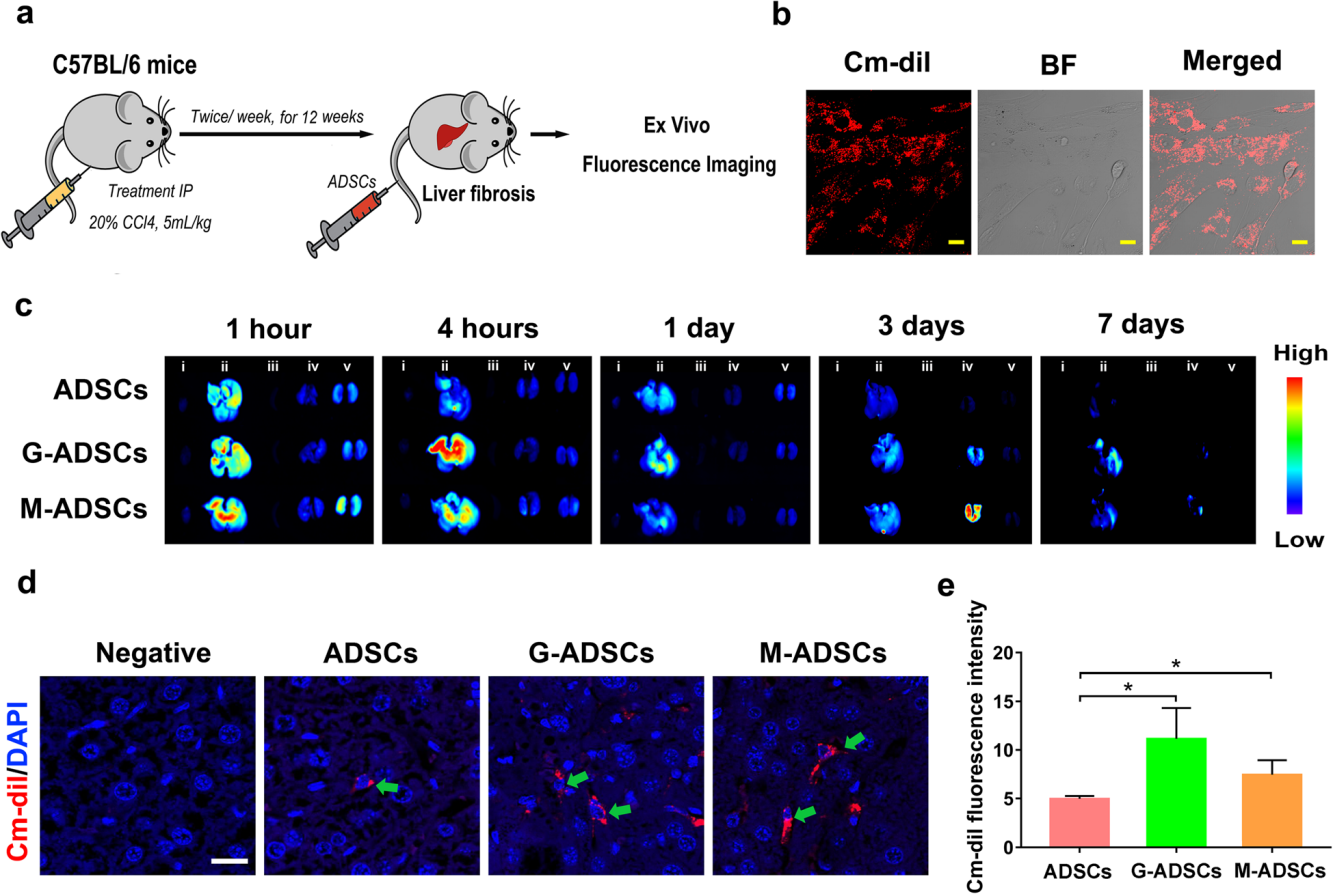
Antioxidant preconditioning increases the intrahepatic engraftment of ADSCs in vivo. **a** Schematic illustration of the intravenous administration of Cm-dil labeled ADSCs with or without antioxidant pretreatment. **b** Representative images of Cm-dil labeled ADSCs (scale bar, 20 μm). **c** The distribution of ADSCs in major organs including the heart (i), liver (ii), spleen (iii), lung (iv), and kidney (v) after cell transplantation for 1 h, 4 h, 1 day, 3 days, and 7 days, respectively. **d** The hepatic retention of ADSCs after cell transplantation for 7 days (scale bar, 20 μm). **e** The fluorescence intensity of ADSCs after cell transplantation for 7 days (*n* = 3 per group; *p < 0.05). ADSCs, adipose tissue-derived mesenchymal stem cells; G-ADSCs, ADSCs pretreated with reduced glutathione; M-ADSCs, ADSCs pretreated with melatonin

In order to investigate the effects of antioxidant preconditioning on hepatic engraftment efficiency of ADSCs, the ADSCs were labeled with Cm-dil and subsequently transplanted into the liver fibrosis mice *via* tail vein injection. Then, the major organs including the heart, liver, spleen, lung, and kidney were collected for ex vivo imaging after ADSC transplantation for 1 h, 4 h, 1 day, 3 days, and 7 days, respectively (Fig. 3a). The results showed that the fluorescence dye (Cm-dil) was clearly observed in ADSCs, which means the successful labeling of ADSCs by Cm-dil (Fig. 3b). Comparing with the control mice without antioxidant precondition, remarkably enhanced fluorescence intensity in liver tissues was observed in the groups of GSH- or melatonin-pretreated ADSC transplantation for several time points including 4 h, 1 day, 3 days, and 7 days, suggesting that the enhanced hepatic engraftment of ADSCs could be achieved by antioxidant preconditioning with GSH or melatonin (Fig. 3c). To further confirm the intrahepatic engraftment of ADSCs, the liver tissues (after ADSC transplantation for 7 days) were paraffin embedded and sectioned into slices to observe the engrafted cells through labeled fluorescence. As shown in Fig. 3d, e, the increased hepatic retention and fluorescence intensity of ADSCs were clearly observed in the antioxidant preconditioning groups comparing with the untreated ADSC group, which further confirmed that the antioxidant preconditioning could promote ADSC retention in the fibrotic liver tissues. Taken together, these data suggested that antioxidant preconditioning could improve the engraft efficiency of ADSC transplantation for liver fibrosis.

### Protective effects of antioxidant preconditioning on oxidative stress-induced cell injury

Since ROS-induced oxidative stress is a major characteristic of liver fibrosis, and it is also the main reason that is responsible for the extremely low engraft efficiency of ADSC transplantation in liver fibrosis [24], the protective effects of antioxidant preconditioning on ROS-induced oxidative cell injury was further investigated. Here, a typical H_2_O_2_-induced cell injury model was applied to mimic the cell oxidative injury to investigate the protective effects of antioxidant pretreatment (including GSH and melatonin) on ROS-induced ADSC oxidative injury.

**Fig. 4:**
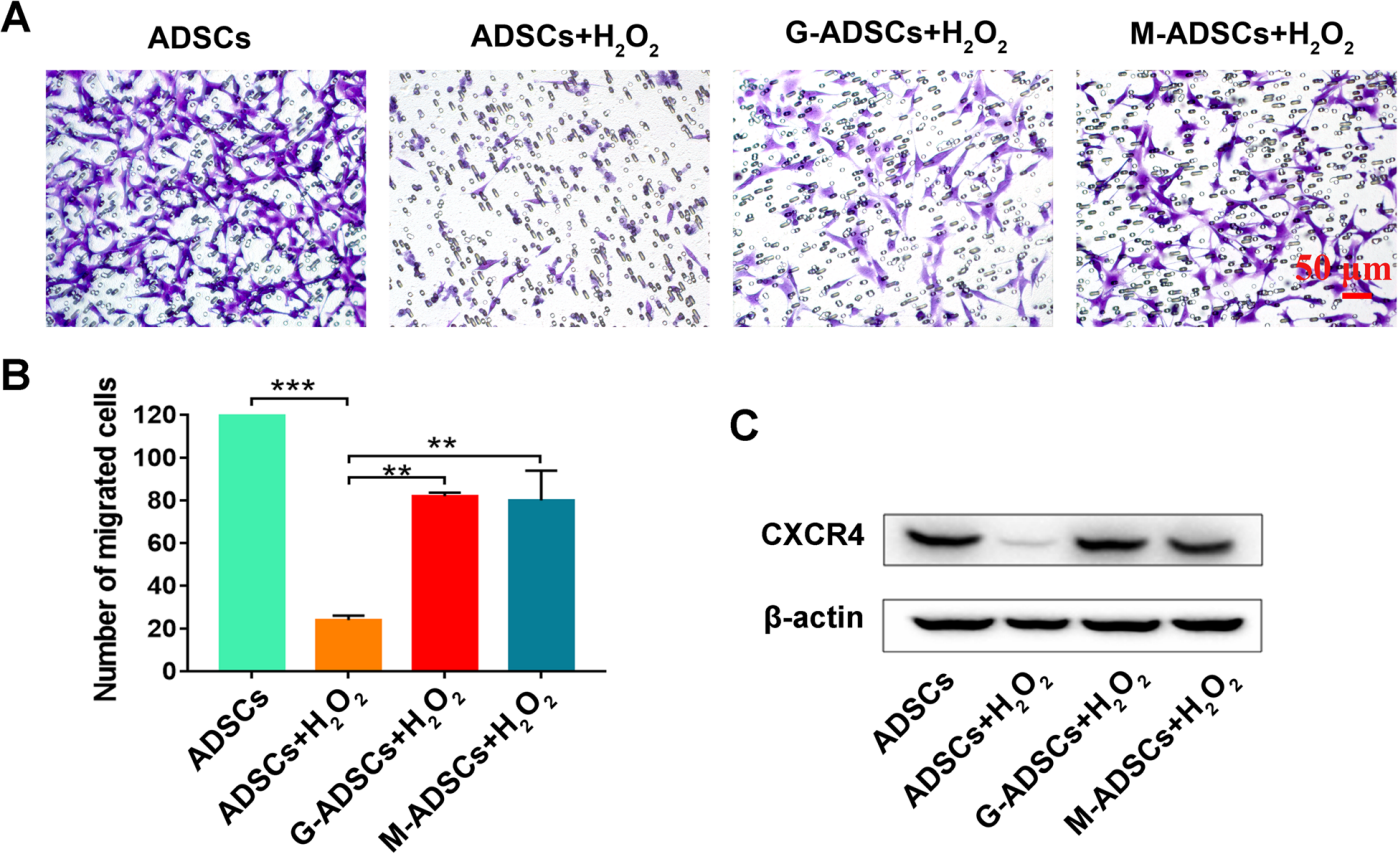
Antioxidant preconditioning promotes the cell migration of ADSCs in vitro. **a** ADSC migration after treatment with H_2_O_2_ (× 200 magnification; scale bar, 50 μm). **b** Quantification of ADSC migration after treatment with H_2_O_2_ (*n* = 5 per group; **p < 0.01; ***p < 0.001). **c** Western blotting of CXCR4 in ADSCs injured by H_2_O_2_. ADSCs, adipose tissue-derived mesenchymal stem cells; G-ADSCs, ADSCs pretreated with reduced glutathione; M-ADSCs, ADSCs pretreated with melatonin; H_2_O_2_, hydrogen peroxide; CXCR4, C-X-C chemokine receptor type 4

Considering that the increased intrahepatic engraftment of ADSCs might due to the protective effect of antioxidant preconditioning on enhanced cell migration ability of ADSCs under oxidative stress environment, we next investigated the effect of antioxidant precondition on cell migration of ADSCs under H_2_O_2_ treatment. As shown in Fig. 4a, b, the number of migrated ADSCs was significantly decreased in the H_2_O_2_-treated group compared with the normal ADSCs, thus indicating the inhibited cell migration of ADSCs induced by oxidative injury, while the cell migration capacity could be improved in the GSH- or melatonin-pretreated group compared with the un-pretreated ADSCs under the oxidative-injured condition, suggesting that antioxidant preconditioning could improve the cell migration ability of ADSCs against oxidative injury; additionally, similar results were also observed using the wound healing assay (Fig. S1). To further confirm the protective effect of antioxidant preconditioning on ADSC migration and homing, the cell adhesion assay was also performed. As shown in Fig. S2, the number of adhered ADSCs was significantly decreased in the H_2_O_2_-treated group compared with the normal ADSCs, thus indicating the inhibited cell adhesion of ADSCs induced by oxidative injury, while the cell adhesion capacity of ADSCs could be partially recovered in the GSH- or melatonin-pretreated group compared with the un-pretreated cells under the oxidative-injured condition, suggesting that antioxidant preconditioning could protect the ADSC adhesion ability against oxidative injury.

Since CXCR4 has been reported to be one of key chemokine receptor which can mediate stem cell migration and homing to the targeted sites [25], we next analyzed the protein expression of CXCR4 in ADSCs injured by H_2_O_2_. Our results have shown that the decreased expression of CXCR4 in ROS-injured ADSCs could be reversed by using the pretreatment of GSH or melatonin, which suggested that antioxidant preconditioning could enhance CXCR4 expression of ADSCs under oxidative injury condition. Overall, these results demonstrated that antioxidant preconditioning could promote cell migration ability of ADSCs in the oxidative condition.

**Fig. 5:**
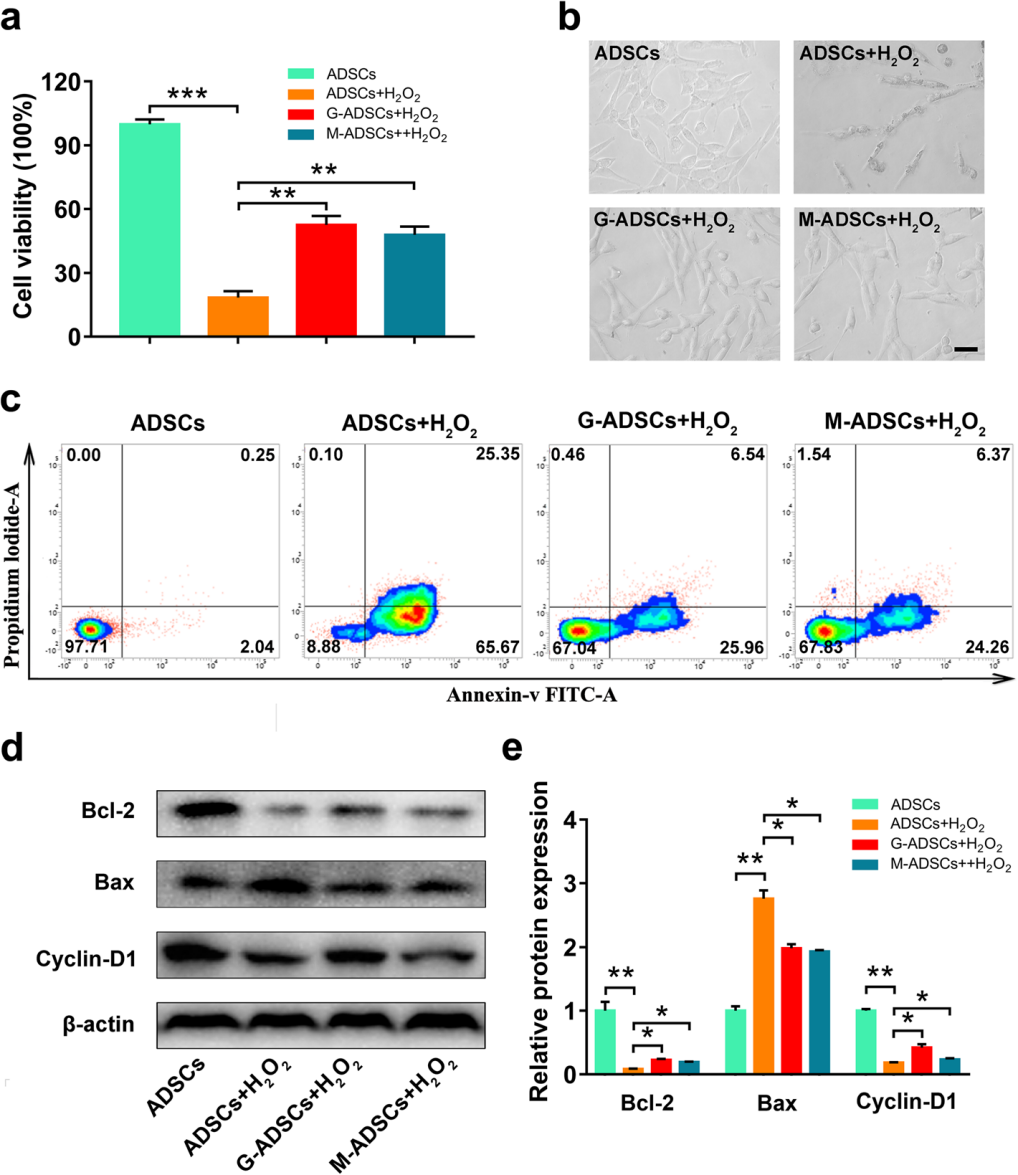
Antioxidant preconditioning promotes the cell survival of ADSCs in vitro. **a** Cell viability of ADSCs with or without antioxidant pretreatment in H_2_O_2_-induced cell injury model (*n* = 5 per group; **p < 0.01; ***p < 0.001). **b** Representative images of cell morphology of ADSCs treated with H_2_O_2_ (scale bar, 50 μm). **c** Cell apoptosis was evaluated by flow cytometry. **d** Western blotting of Bcl-2, Bax, and Cyclin-D1 in ADSCs treated with H_2_O_2_. **e** Relative expression of Bcl-2, Bax, and Cyclin-D1 in ADSCs treated with H_2_O_2_ (*n* = 3 per group; *p < 0.05; **p < 0.01). ADSCs, adipose tissue-derived mesenchymal stem cells; G-ADSCs, ADSCs pretreated with reduced glutathione; M-ADSCs, ADSCs pretreated with melatonin; H_2_O_2_, hydrogen peroxide

Next, we investigated the protective effects of antioxidant preconditioning on cell survival of ADSCs. As shown Fig. 5a–c, the decreased cell viability and the increased cell apoptosis of ADSCs were obviously observed in the H_2_O_2_-treated group compared with those in normal ADSCs, which means the decreased cell survival rate of ADSCs in the oxidative-injured environment; however, these cell injuries could be significantly inhibited after antioxidant preconditioning with GSH or melatonin compared with the un-pretreated ADSCs, suggesting that antioxidant preconditioning could promote cell viability and inhibit cell apoptosis under the oxidative-injured condition. Additionally, we also examined the key makers of cell proliferation and apoptosis to further show the protective mechanisms of antioxidant preconditioning on ADSC cell survival. As shown in Fig. 5d, e, antioxidant pretreatment significantly increased the expression of Bcl-2 and Cyclin-D1, and downregulated the Bax expression, thus indicating that antioxidant preconditioning could enhance the cell survival of ADSCs by promoting cell proliferation and inhibiting cell apoptosis.

Taken together, these data suggested that the enhanced hepatic engraftment of ADSCs might partly attribute to the protective effects of antioxidant preconditioning on cell migration and cell survival of ADSCs within the oxidative stress environment.

## Discussion

The low cell engraftment efficiency always leads to ADSC therapy failure, which is becoming a major obstacle hindering clinical applications of ADSCs. In this study, we develop a novel strategy based on antioxidant preconditioning including GSH and melatonin to enhance intrahepatic engraftment of ADSC transplantation for liver fibrosis. By low dose of GSH or melatonin pretreatment on ADSCs before transplantation, we certified that this strategy (antioxidant preconditioning) could promote therapeutic functions of ADSCs for liver fibrosis in vivo. Particularly, we also found the increased hepatic retention of ADSCs in fibrotic liver parenchyma after ADSC transplantation for 7 days. Hence, our study provides a novel strategy for enhancing ADSC therapy of liver fibrosis.

The high level of ROS seriously impairs stem cell functions including migration, adhesion, and proliferation, as well as the stemness and multiple differential potential, finally leading to low cell engraftment efficiency in vivo [22, 26, 27]. To overcome this obstacle, antioxidant preconditioning has been previously used to improve ADSC therapy for other diseases, including acute lung ischemia-reperfusion injury [27], sepsis-induced kidney injury [28], and acute interstitial cystitis [29]. In particular, melatonin pretreatment has been used to improve the hepatic homing and therapy of bone marrow-derived mesenchymal stem cells (BMSCs) in liver fibrosis [30, 31]. However, recent evidence has been suggested that melatonin pretreatment could be more applicable in enhancing cell therapy of ADSCs than in BMSCs [32], and hence, it is worthwhile to apply melatonin pretreatment to improve the therapeutic effect of ADSCs for liver fibrosis. As predicted, we also found that melatonin pretreatment significantly increased the hepatic retention of ADSCs and promoted ADSC therapy for CCl_4_-injured liver fibrosis in the current study. Considering glutathione is an important endogenous antioxidant for protecting stem cell growth [33] and GSH is a major drug for treating liver diseases [34], GSH was also used for the pretreatment of ADSC therapy for liver fibrosis in our study. Of note, compared with melatonin pretreatment, we found the hepatic engraftment of ADSCs could be further improved by using GSH pretreatment (after ADSC transplantation for 4 h and 7 days, respectively). Therefore, our data suggested that GSH pretreatment might be more suitable for enhancing ADSC therapy for liver fibrosis.

Given that oxidative stress, as a typical characteristic of liver fibrosis, is a major issue in the low survival rate of ADSC transplantation, we further investigated the protective effect of antioxidant preconditioning on a typical oxidative-injured model (H_2_O_2_ induction) in vitro. As predicted, we found that antioxidant preconditioning could promote cell viability and inhibit cell apoptosis in H_2_O_2_-injured ADSCs, which in turn revealed the mechanisms that antioxidant preconditioning enhanced ADSC survival rate through reducing oxidative stress induced by ROS. Accordingly, targeted regulation of oxidative stress in injured liver (in vivo) might be another strategy for enhancing stem cell engraftment, and the relevant work deserves further study.

Here, we demonstrated that antioxidant preconditioning could effectively enhance transplantation efficiency and therapeutic outcomes of ADSCs for liver fibrosis, and this finding might be a promising and practical approach for accelerating clinic translation of ADSC-based therapy.

## Conclusion

In conclusion, we demonstrated that antioxidant preconditioning could effectively enhance the intrahepatic engraftment efficiency of ADSCs, therefore improving the therapeutic effect of ADSC therapy for liver fibrosis, and antioxidant preconditioning might present a practical strategy for enhancing ADSC therapy.

## Supplementary information

**Additional file 1: Figure S1.** Antioxidant preconditioning promotes cell motility of ADSCs in vitro. **a** ADSC motility after treatment with 100 μM H_2_O_2_ (×100 magnification; scale bar, 100 μm). **b** Quantification of ADSC migration rate after treatment with H_2_O_2_ (*n* = 5 per group; **p < 0.01; ***p < 0.001). ***ADSCs*** adipose tissue-derived mesenchymal stem cells, ***G-ADSCs*** ADSCs pretreated with reduced glutathione, ***M-ADSCs*** ADSCs pretreated with melatonin, ***H***_***2***_***O***_***2***_ hydrogen peroxide.

**Additional file 2: Figure S2.** Antioxidant preconditioning promotes cell adhesion of ADSCs in vitro. **a** ADSC adhesion after treatment with 300 μM H_2_O_2_ (×200 magnification; scale bar, 50 μm). **b** Quantification of number of adhesive ADSCs after treatment with H_2_O_2_ (n = 5 per group; *p < 0.05; ***p < 0.001). ***ADSCs*** adipose tissue-derived mesenchymal stem cells, ***G-ADSCs*** ADSCs pretreated with reduced glutathione, ***M-ADSCs*** ADSCs pretreated with melatonin, ***H***_***2***_***O***_***2***_ hydrogen peroxide.

**Additional file 3: Supplementary materials.**

## Data Availability

The data sets supporting the results of this article are included within the article.

## Abbreviations

ADSCs: Adipose tissue-derived mesenchymal stem cells
ROS: Reactive oxygen species
GSH: Reduced glutathione
CCl_4_: Carbon tetrachloride
AST: Aspartate aminotransferase
ALT: Alanine aminotransferase
TBIL: Total bilirubin
ALB: Albumin
PFA: Paraformaldehyde
H_2_O_2_: Hydrogen peroxide
CXCR4: C-X-C chemokine receptor type 4
