# Supplementary figures and images for: Antioxidant preconditioning improves therapeutic outcomes of adipose tissue-derived mesenchymal stem cells through enhancing intrahepatic engraftment efficiency in a mouse liver fibrosis model
Source: Stem Cell Res Ther. 2020 Jun 16;11:237. doi: 10.1186/s13287-020-01763-y (PMC7298967; doi:10.1186/s13287-020-01763-y)

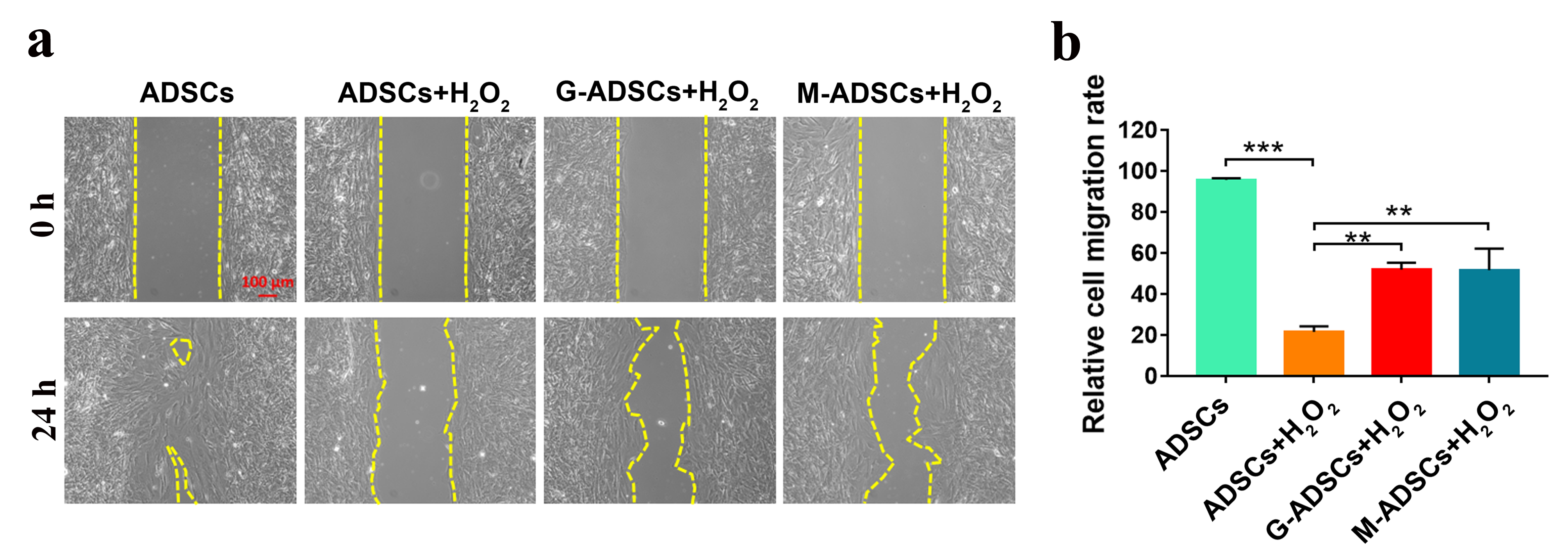

Supplement: Supplementary file 1 — Additional file 1: Figure S1. Antioxidant preconditioning promotes cell motility of ADSCs in vitro. a ADSC motility after treatment with 100 μM H2O2 (×100 magnification; scale bar, 100 μm). b Quantification of ADSC migration rate after treatment with H2O2 (n = 5 per group; **p < 0.01; ***p < 0.001). ADSCs adipose tissue-derived mesenchymal stem cells, G-ADSCs ADSCs pretreated with reduced glutathione, M-ADSCs ADSCs pretreated with melatonin, H2O2 hydrogen peroxide. [file 13287_2020_1763_MOESM1_ESM.tif]

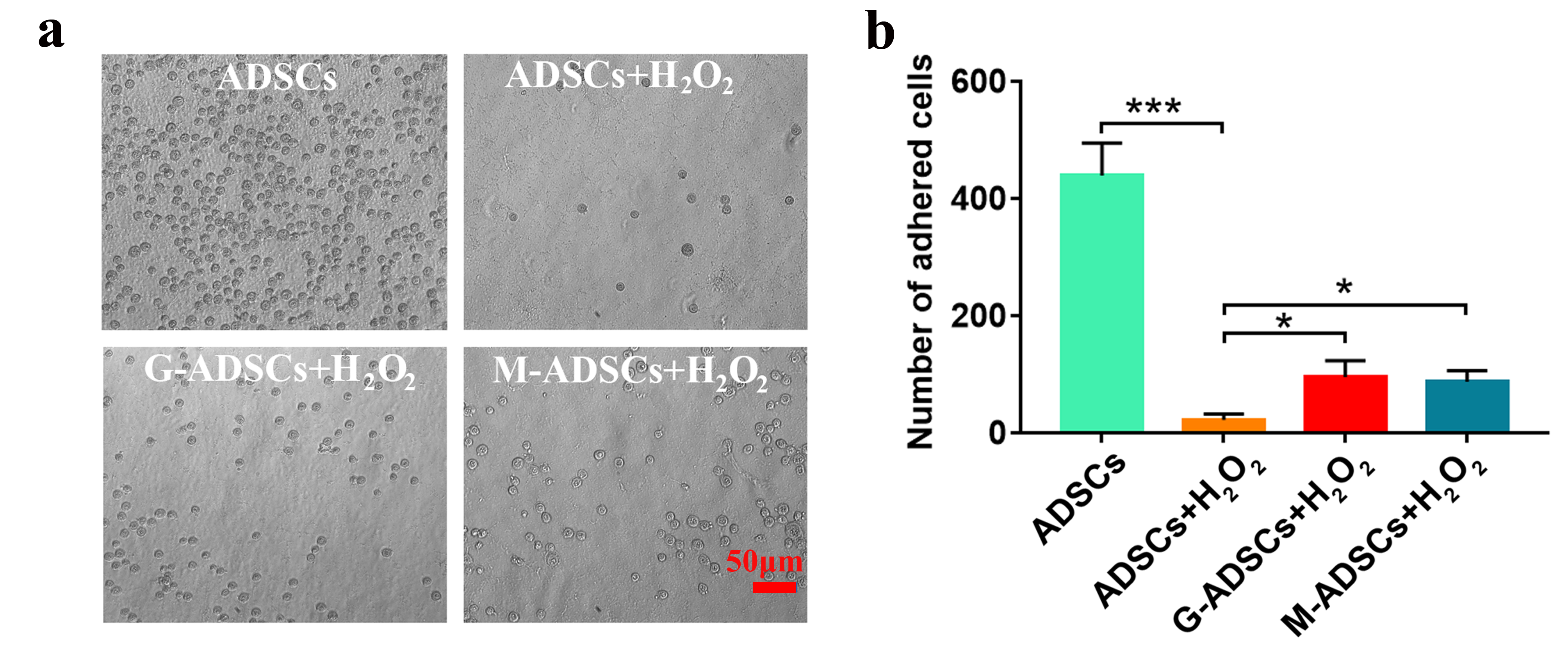

Supplement: Supplementary file 2 — Additional file 2: Figure S2. Antioxidant preconditioning promotes cell adhesion of ADSCs in vitro. a ADSC adhesion after treatment with 300 μM H2O2 (×200 magnification; scale bar, 50 μm). b Quantification of number of adhesive ADSCs after treatment with H2O2 (n = 5 per group; *p < 0.05; ***p < 0.001). ADSCs adipose tissue-derived mesenchymal stem cells, G-ADSCs ADSCs pretreated with reduced glutathione, M-ADSCs ADSCs pretreated with melatonin, H2O2 hydrogen peroxide. [file 13287_2020_1763_MOESM2_ESM.tif]
