# Supplementary material for: Antioxidant preconditioning improves therapeutic outcomes of adipose tissue-derived mesenchymal stem cells through enhancing intrahepatic engraftment efficiency in a mouse liver fibrosis model
Source: Stem Cell Res Ther. 2020 Jun 16;11:237. doi: 10.1186/s13287-020-01763-y (PMC7298967; doi:10.1186/s13287-020-01763-y)
Supplement: Supplementary file 3 — Additional file 3: Supplementary materials. [file 13287_2020_1763_MOESM3_ESM.doc]

**Antioxidant preconditioning improves therapeutic outcomes of adipose tissue-derived mesenchymal stem cells through enhancing intrahepatic engraftment efficiency in a mouse liver fibrosis model**

Naishun Liao^1,2,3^, Yingjun Shi^1,2,3^, Yingchao Wang^1,2,3^, Fangyu Liao^1,2,3^, Bixing Zhao^1,2,3^, Youshi Zheng^1,2,3^, Yongyi Zeng^1,2,3,4^, Xiaolong Liu^1,2,3*^ and Jingfeng Liu^1,2,3, 4*^

^1^ The United Innovation of Mengchao Hepatobiliary Technology Key Laboratory of Fujian Province, Mengchao Hepatobiliary Hospital of Fujian Medical University, Fuzhou 350025, P.R. China

^2^ Liver Disease Center, The First Affiliated Hospital of Fujian Medical University, Fuzhou 350007, P.R. China

^3^ Mengchao Med-X Center, Fuzhou University, Fuzhou 350116, P.R. China

^4^ The Liver Center of Fujian Province, Fujian Medical University, Fuzhou 350025, P.R. China

**Corresponding authors: correspondence should be address to* Prof Xiaolong Liu and Prof Jingfeng Liu. E-mail: xiaoloong.liu@gmail.com and drjingfeng@126.com.

**Supplementary Material**

**Scratch wound healing assay**

ADSCs at a density of 1.0 × 10^5^ cells/well were respectively seeded into a 35-mm culture-insert dish (Ibidi, Germany) according to the manufacture’ introduction. 24 hours later, the plugin was removed leaving a cell-free gap (about 500 μm), and then the cultured medium of each well was replaced by fresh complete medium supplied with 100 μM H_2_O_2_, and the cells incubated with complete medium were used as the negative control. After incubation for 24 hours, the migration rate into this “wond area” was observed and measured using a Carl Zeiss microscope (Zeiss, Germany).

**Cell adhesion assay**

For observation the effect of antioxidant preconditioning on ADSC adhesion under oxidative condition, 96-well microplates were pretreated with 50 μL matrigel (20 μg/mL, BD, USA) at 4 ℃ overnight. After that, the pretreated 96-well plates were washed with 0.01M PBS for 3 times, and the cells at a density of 5.0 × 10^3^ cells/well were re-suspended with complete medium supplied with 300 μM H_2_O_2_ and seeded into the plates. 30 minutes later, the attached cells were fixed in absolute methanol. Afterwards, the cells were washed with PBS and observed using a Carl Zeiss microscope (Zeiss, Germany).
